# Safety and Efficacy of Sofosbuvir with Ribavirin® in Hepatitis C, Genotype 3 Patients with Cirrhosis: A Real-world Experience

**DOI:** 10.7759/cureus.4012

**Published:** 2019-02-04

**Authors:** Nazish Butt, Ali Akbar, Amanullah Abbasi, Sehrish Reema, Jaffer Bin Baqar, Qurban Hussain Shaikh

**Affiliations:** 1 Gastroenterology, Jinnah Postgraduate Medical Centre, Karachi, PAK; 2 Internal Medicine, Jinnah Postgraduate Medical Center, Karachi, PAK; 3 Internal Medicine, Dow University of Health Sciences, Karachi, PAK; 4 Internal Medicine, Jinnah Postgraduate Medical Centre, Karachi, PAK; 5 Miscellaneous, University of Karachi, Karachi, PAK

**Keywords:** cirrhosis, gt-3, sofosbuvir, ribavirin

## Abstract

Introduction

Hepatitis C virus (HCV) is the leading cause of cirrhosis. The advent of Directly Acting Antivirals (DAAs) like Sofosbuvir (SOF) has dramatized the treatment and is the cornerstone for the treatment of HCV. Most trials have been conducted in HCV genotype 1 (GT-1) and data for Interferon-free regimen in genotype 3 (GT-3) is limited especially in cirrhotic patients.

Aim

To evaluate the safety and efficacy of SOF plus Ribavirin® (RIB) in patients with compensated and decompensated cirrhosis.

Methods

This was a quasi-experimental study in HCV patients with compensated and decompensated cirrhosis. Each group (compensated and decompensated) was further subdivided into the treatment-naïve and treatment-experienced groups. Efficacy was assessed by end treatment response (ETR) and sustained viral response (SVR) in the treatment-naïve and experienced groups. Adverse events were recorded on designed proforma on serial follow-up visits.

Results

The study consisted of 110 consecutive patients. Among 110 patients, 51 had compensated cirrhosis and 59 had decompensated cirrhosis. The mean age was 53.8 ± 11 years. Males were n=56 (50.9%) and females were n=54 (49.1%). All the patients in Child-Turcotte-Pugh class A were in the compensated group. CTP B class was found to be 10.5% and 89.5% in the compensated and decompensated groups, respectively, whereas all the patients in CTP class C were in the decompensated group.

In the compensated cirrhosis group, ETR was achieved in 36 (87.8%) treatment-naïve and 8 (88.9%) experienced patients. In decompensated cirrhosis, treatment-naïve and experienced patients achieved ETR in 28 (82.4%) and 18 (85.7%) patients, respectively. Whereas in compensated cirrhosis treatment-naïve and experienced patients, SVR was achieved in 25 (83.3%) and five (71.4%), respectively. In decompensated cirrhosis, 21 (77.8%) treatment-naïve and 12 (75%) experienced patients achieved SVR.

The most common adverse events experienced by the patients were fatigue followed by myalgia, nausea, and diarrhea. The new onset of complications found due to cirrhosis were ascites, followed by hepatoma, upper gastrointestinal bleed, portosystemic encephalopathy, acute on chronic liver failure, and death.

Conclusion

Sofosbuvir in combination with Ribavirin® is safe but suboptimal in treatment outcomes, particularly in treatment-experienced patients with decompensated cirrhosis than in treatment-naive patients with compensated cirrhosis due to HCV GT-3.

## Introduction

Hepatitis C virus (HCV) is a significant health problem worldwide and affects 170 million people [1]. Chronic infection by this virus causes liver fibrosis, resulting in end-stage liver disease and hepatocellular carcinoma [2]. The successful eradication of the virus by treatment leads to sustained viral response (SVR), which is linked with a decrease in all-cause mortality [3-4]. Before the advent of direct-acting antivirals (DAAs), the only treatment option was Interferon-based regimens with an insufficient response, poor compliance, and troublesome adverse effects. In the recent past, DAAs have shifted the landscape of HCV management [5], with sofosbuvir (SOF) as the backbone.

SOF is a pan-genotypic nucleotide analog that acts as an inhibitor of NS5B polymerase [6-7]. Genotype 3 (GT-3) HCV always showed different results in comparison to other genotypes with various studies, elaborating its relationship with early fibrosis and the development of hepatocellular carcinoma [8]. In contrast to genotype 1 (GT-1) and genotype 2, GT-3-infected patients' response is low, with the best at 80% to 85% with all oral drug regimens [9].

Globally, Pakistan stands second in HCV infections, with predominant GT-3 accounting for 79% of HCV infections [10]. A great bulk of international research is already done especially from the west with an emphasis on GT-1. Data of GT-3-related liver cirrhosis is scanty. Therefore, this study will look into the efficacy of SOF-based therapy in this part of the world, particularly in patients with HCV GT-3-associated liver cirrhosis.

## Materials and methods

This was a quasi-experimental study conducted in the gastroenterology section of Medical Unit IV, Jinnah Postgraduate Medical Centre, Karachi, and Medical Unit II, Dow University Hospital, Ojha Campus, Karachi, after the approval of the institutional ethical committee. Patients were enrolled from March 2016 to September 2017 after the written informed consent of the participants.

Patients

All patients aged 18 years or older who were compensated or decompensated cirrhotics with HCV ribonucleic acid (RNA) detected by polymerase chain reaction (PCR), with a lower limit of 15 IU/ml, and GT-3 were included. Decompensated cirrhosis was defined by prior or recent findings of ascites, hepatic encephalopathy, variceal hemorrhage, along with ultrasound findings of shrunken liver with irregular margins, splenomegaly with or without evidence of ascites, and F3/F4 stage on Fibroscan® (Echosens, Paris, France).

In contrast, those patients who had no previous or current evidence of ascites, encephalopathy, and hematemesis with evidence of cirrhosis on ultrasound and Fibroscan® were labelled as compensated cirrhosis. Patients with uncontrolled diabetes mellitus, uncontrolled hypertension, unstable heart failure, stroke, eGFR<30 ml/min, hepatocellular carcinoma, and tuberculosis were excluded.

Treatment

SOF and Ribavirin® (RIB) treatment was given to all participants for the period of 24 weeks, irrespective of treatment-naïve and treatment-experienced with Interferon. Treatment-experienced patients included relapsers and non-responders. Relapse was defined as the reappearance of HCV RNA on PCR after the completion of treatment whereas nonresponders included those patients who failed to clear the HCV RNA in blood after 24 weeks of treatment. Patients were offered single oral 400 mg of SOF on a daily basis and weight-based oral RIB (1000 mg if weight <75 kg, 1200 mg if weight > 75 kg) in two divided doses. Follow-up of patients was done for at least 24 weeks after the completion of treatment.

Study assessments

Baseline data collection included demographic data, previous history of HCV treatment, laboratory investigations, including serum hemoglobin (Hb), platelet count, total bilirubin, alanine transaminase (ALT), aspartate transaminase (AST), albumin, creatinine, prothrombin time (PT), international normalized ratio (INR), HCV RNA, HCV genotype, ultrasound, alpha-fetoprotein, and Fibroscan®. The severity of cirrhosis was calculated by model of end-stage liver disease (MELD) and Child-Turcotte-Pugh (CTP) scores.

Primary outcome

The efficacy of the regimen was documented by negative/undetectable levels of HCV RNA on PCR quantitative analysis after 24 weeks of treatment, i.e., end of treatment response (ETR). Furthermore, the patients were followed up to assess the complete eradication of the virus via a PCR quantitative analysis to document sustained virological response (SVR) at 24 weeks post-treatment.

Secondary outcome

The safety of the regimen was seen in the light of adverse events occurring that could be solely attributed to the DAAs. All adverse events were documented throughout the time the patient was taking the medications and during follow-up.

Statistical analysis

Data analysis was performed by using SPSS 22.0 software (Chicago, IL, USA). The primary endpoints were ETR and SVR. Primary analysis was conducted on all those patients who participated in the study and completed the 24 weeks of the regimen. The secondary endpoint was also analyzed. Demographics of patients were compared by using the student's t-test or the Mann-Whitney U test, whichever was suitable. Categorical variables were analyzed by the Chi-square test. A p-value less than 0.05 was considered statistically significant.

## Results

The baseline characteristics of the study population are presented in Table 1. Most patients, 59 (53.63%), had decompensated cirrhosis. Age and gender were comparable for both groups. Patients in the decompensated group had an advanced CTP class and low platelet counts. The rest of the baseline parameters were comparable.

**Table 1:** Baseline characteristics of study population CTP class, Child-Turcotte-Pugh class; Hb, hemoglobin; MCV, mean corpuscular volume; PLT, platelets; TLC, total leucocyte count; PT, prothrombin time; INR, International normalized ratio; TB, total bilirubin; DB, direct bilirubin; ALT, alanine transaminase; AST, aspartate transaminase; ALP, alkaline phosphatase; GGT, gamma-glutamyl transferase; Na, sodium; K, potassium

Variables		Compensated	Decompensated
Age (years)		52.31±13.01	53.85±8.92
Age (years)	Male	53.75±10.16	
	Female	52.50±11.83	
Gender, n (%)	Male	28 (50.0)	28 (50.0)
	Female	23 (42.6)	31 (57.4)
	Total	51 (46.4)	59 (53.6)
CTP Class, n (%)	A	45 (100.0)	0 (0.0)
	B	6 (10.5)	51 (89.5)
	C	0 (0.0)	8 (100.0)
Hb (gm/dl)		10.62±1.20 Gm/dl	8.69±1.12 m/dl
MCV ( Fl )		83.15±12.04	86.48±7.68
PLT (10^9^/L)		112.57±20.67	82.81±23.18
TLC (10^9^/L)		7.54±2.98	6.31±2.53
PT (sec)		12.89±3.32	13.68±3.10
INR		1.08±0.16	1.24±0.33
TB (mg/dl)		0.86±0.39	1.36±0.84
DB (mg/dl)		0.43±0.26	1.80±4.97
ALT (IU/L)		47.98±14.25	42.39±12.09
AST (IU/L)		64.08±74.06	36.32±17.94
ALP (IU/L)		149.86±97.51	195.17±122.72
GGT (IU/L)		116.04±125.64	128.37±233.55
Serum Albumin (gm/dl)		2.98±0.24	2.78±0.30
Urea (mg/dl)		33.84±22.19	44.58±22.24
Creatinine (mg/dl)		0.75±0.27	0.81±0.42
Na (mEq/L)		135.76±4.52	135.68±3.79
K (mEq/L)		4.09±0.43	3.89±0.42

Treatment response

Treatment response, ETR and SVR either achieved or not achieved, were compared in the compensated and decompensated groups. There was no association of ETR and SVR with respect to the compensated and decompensated groups. ETR and SVR were further observed based on gender and age, as shown in Table 2.

**Table 2:** Treatment response in compensated and decompensated patients

				Cirrhosis		P-Value
				Compensated	De-Compensated	
Virological Response	End Treatment Response	Achieved		44 (48.9%)	46 (51.1%)	0.523
		Not Achieved		6 (40.0%)	9 (60.0%)	
	Sustained Virological Response	Achieved		37 (50.7%)	36 (49.3%)	0.48
		Not Achieved		7 (41.2%)	10 (58.8%)	
Virological Response	End Treatment Response	Achieved	Male	24 (54.5%)	20 (45.5%)	0.29
			Female	20 (43.5%)	26 (56.5%)	
		Not Achieved	Male	4 (50.0%)	4 (50.0%)	0.61
			Female	2 (28.6%)	5 (71.4%)	
		Total	Male	28 (53.8%)	24 (46.2%)	0.20
			Female	22 (41.5%)	31 (58.5%)	
	Sustained Virological Response	Achieved	Male	23 (62.2%)	14 (37.8%)	0.05
			Female	14 (38.9%)	22 (61.1%)	
		Not Achieved	Male	1 (14.3%)	6 (85.7%)	0.13
			Female	6 (60.0%)	4 (40.0%)	
		Total	Male	24 (54.5%)	20 (45.5%)	0.29
			Female	20 (43.5%)	26 (56.5%)	
Virological Response	End Treatment Response	Achieved	<= 50	26 (55.3%)	21 (44.7%)	0.20
			>50	18 (41.9%)	25 (58.1%)	
		Not Achieved	<= 50	2 (40.0%)	3 (60.0%)	1.00
			>50	4 (40.0%)	6 (60.0%)	
		Total	<= 50	28 (53.8%)	24 (46.2%)	0.20
			>50	22 (41.5%)	31 (58.5%)	
	Sustained Virological Response	Achieved	<= 50	20 (52.6%)	18 (47.4%)	0.73
			>50	17 (48.6%)	18 (51.4%)	
		Not Achieved	<= 50	6 (66.7%)	3 (33.3%)	0.05
			>50	1(12.5%)	7 (87.5%)	
		Total	<= 50	26 (55.3%)	21 (44.7%)	0.21
			>50	18 (41.9%)	25 (58.1%)	

The gender of the patient was found to be a significant factor affecting the primary outcomes. Males were more likely to achieve SVR in the compensated cirrhotics group, whereas females were more likely to achieve SVR in the decompensated cirrhotics group. This was demonstrated by a significant p-value of 0.05.

On the basis of age, patients were divided into two age groups, ≤50 and >50, and their treatment response was noted. Patients aged >50 years with decompensated cirrhosis had lower rates of SVR. However, patients aged ≤50 years with compensated cirrhosis achieved higher rates of SVR. This resulted in a significant p-value of 0.05, as summarized in Table 2.

Response in Treatment-naive Patients

Treatment response was also observed in treatment-naïve compensated and treatment-naïve decompensated, as shown in Table 3. There was no association with respect to treatment groups in ETR (p-value=0.61) and SVR (p-value=0.21).

**Table 3:** Response in treatment-naive patients

			Treatment group		p-value
			Treatment-naive compensated	Treatment-naive decompensated	
End Treatment Response	Achieved		36 (53.7%)	31 (46.3%)	0.61
	Not Achieved		5 (45.5%)	6 (54.5%)	
Sustained Virological Response	Achieved		32 (57.1%)	24 (42.9%)	0.21
	Not Achieved		4 (36.4%)	7 (63.6%)	
End Treatment Response	Achieved	Male	23 (57.5%)	17 (42.5%)	0.45
		Female	13 (48.1%)	14 (51.9%)	
	Not Achieved	Male	3 (50.0%)	3 (50.0%)	1.00
		Female	2 (40.0%)	3 (60.0%)	
	Total	Male	26 (56.5%)	20 (43.5%)	0.40
		Female	15 (46.9%)	17 (53.1%)	
Sustained Virological Response	Achieved	Male	22 (64.7%)	12 (35.3%)	0.15
		Female	10 (45.5%)	12 (54.5%)	
	Not Achieved	Male	1 (16.7%)	5 (83.3%)	0.24
		Female	3 (60.0%)	2 (40.0%)	
	Total	Male	23 (57.5%)	17 (42.5%)	0.45
		Female	13 (48.1%)	14 (51.9%)	
End Treatment Response	Achieved	<= 50	20 (62.5%)	12 (37.5%)	0.17
		>50	16 (45.7%)	19 (54.3%)	
	Not Achieved	<= 50	2 (66.7%)	1 (33.3%)	0.54
		>50	3 (37.5%)	5 (62.5%)	
	Total	<= 50	22 (62.9%)	13 (37.1%)	0.10
		>50	19 (44.2%)	24 (55.8%)	
Sustained Virological Response	Achieved	<= 50	17 (63.0%)	10 (37.0%)	0.39
		>50	15 (51.7%)	14 (48.3%)	
	Not Achieved	<= 50	3 (60.0%)	2 (40.0%)	0.24
		>50	1 (16.7%)	5 (83.3%)	
	Total	<= 50	20 (62.5%)	12 (37.5%)	0.17
		>50	16 (45.7%)	19 (54.3%)	

Furthermore, there was no significant difference noted in terms of ETR and SVR based on gender and age in treatment-naive patients in both groups.

Response in Treatment-experienced Patients

Treatment response was also observed in treatment-experienced compensated and treatment-experienced decompensated groups, as depicted in Table 4. There was no association with respect to treatment groups in ETR (p-value=0.70) and SVR (p-value=0.36).

**Table 4:** Response in treatment-experienced patients

			Treatment group		p-value
			Treatment-experienced compensated	Treatment-experienced decompensated	
End Treatment Response	Achieved		8 (34.8%)	15 (65.2%)	0.70
	Not Achieved		1 (25.0%)	3 (75.0%)	
Sustained Virological Response	Achieved		5 (29.4%)	12 (70.6%)	0.36
	Not Achieved		3 (50.0%)	3 (50.0%)	
End Treatment Response	Achieved	Male	1 (25.0%)	3 (75.0%)	1.00
		Female	7 (36.8%)	12 (63.2%)	
	Not Achieved	Male	1 (50.0%)	1 (50.0%)	1.00
		Female	0 (0.0%)	2 (100.0%)	
	Total	Male	2 (33.3%)	4 (66.7%)	1.00
		Female	7 (33.3%)	14 (66.7%)	
Sustained Virological Response	Achieved	Male	1 (33.3%)	2 (66.7%)	1.00
		Female	4 (28.6%)	10 (71.4%)	
	Not Achieved	Male	0 (0.0%)	1 (100.0%)	1.00
		Female	3 (60.0%)	2 (40.0%)	
	Total	Male	1 (25.0%)	3 (75.0%)	1.00
		Female	7 (36.8%)	12 (63.2%)	
End Treatment Response	Achieved	≤ 50	6 (40%)	9 (60%)	0.66
		≥ 50	2 (25%)	6 (75%)	
	Not Achieved	≤ 50	0 (0.0%)	2 (100%)	1.00
		≥ 50	1 (50.0%)	1 (50.0%)	
	Total	≤ 50	6 (35.3%)	11 (64.7%)	1.00
		≥ 50	3 (30.0%)	7 (70.0%)	
Sustained Virological Response	Achieved	≤ 50	3 (27.3%)	8 (72.7%)	1.00
		≥ 50	2 (33.3%)	6 (66.7%)	
	Not Achieved	≤ 50	3 (75.0%)	1 (25.0%)	0.40
		≥ 50	0 (0.0%)	2 (100%)	
	Total	≤ 50	6 (40.0%)	9 (60.0%)	0.66
		≥ 50	2 (25.0%)	6 (75.0%)	

Additionally, there was no significant difference in terms of ETR and SVR based on gender and age in treatment-experienced patients in compensated and decompensated groups.

Adverse events

**Figure 1:**
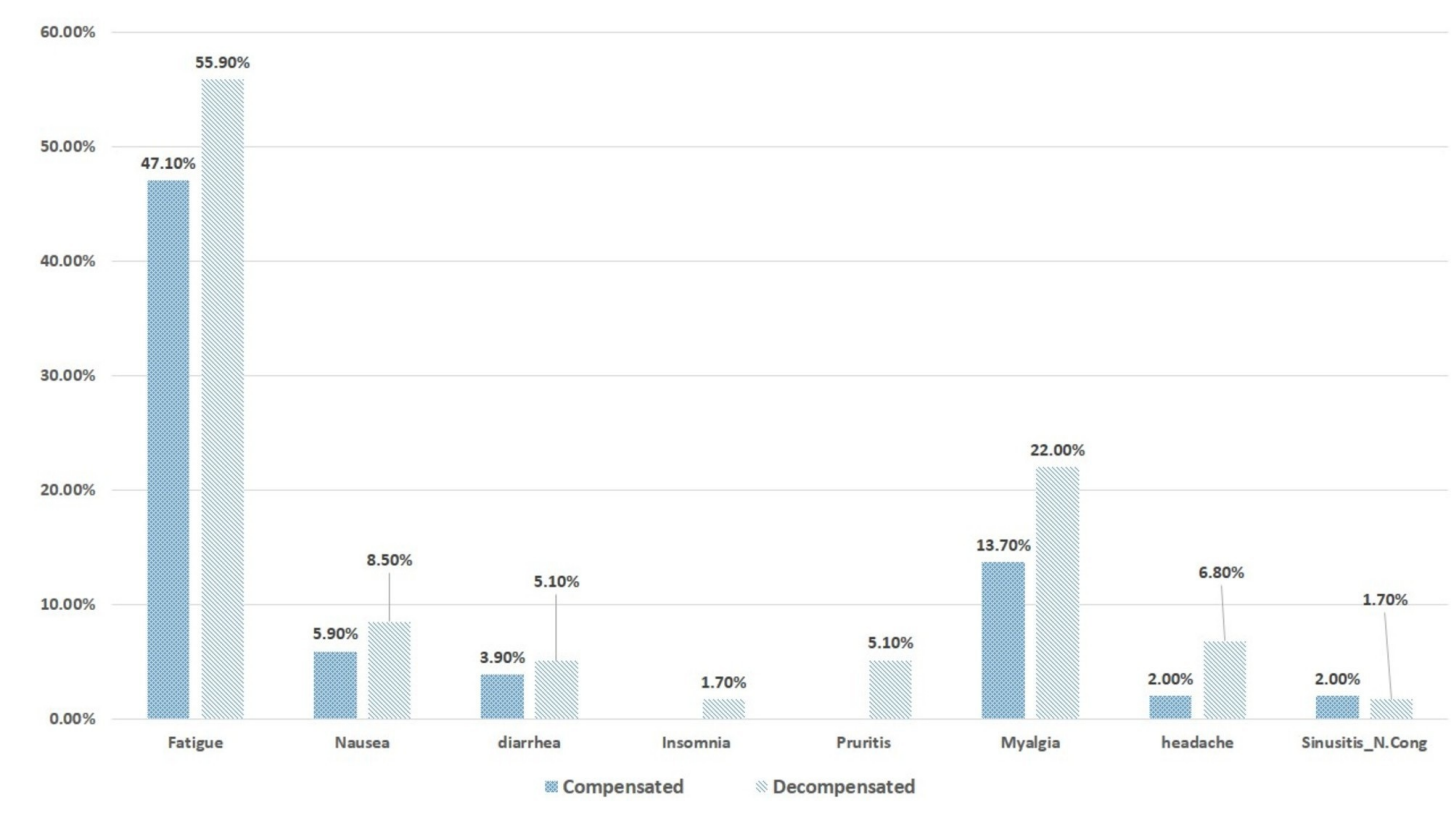
Adverse events in compensated and decompensated patients

Different side effects were noted in the compensated and decompensated groups (Figure 1). Of these, 47.10% have fatigue in the compensated group as compared to 55.90% in the decompensated group; 5.90% of patients in the compensated group complained of nausea as compared to 8.50% in the decompensated group. Diarrhea was present in 3.90% of the patients in the compensated group as compared to 5.10% in the decompensated group. Insomnia and pruritus were present in 1.70% and 5.10% in the decompensated group only. While myalgia, headache, and sinusitis nasal congestion were found to be 13.70%, 2.00%, and 2.00% in the compensated group as compared to 22.00%, 6.80%, and 1.70 % in the decompensated group.

**Figure 2:**
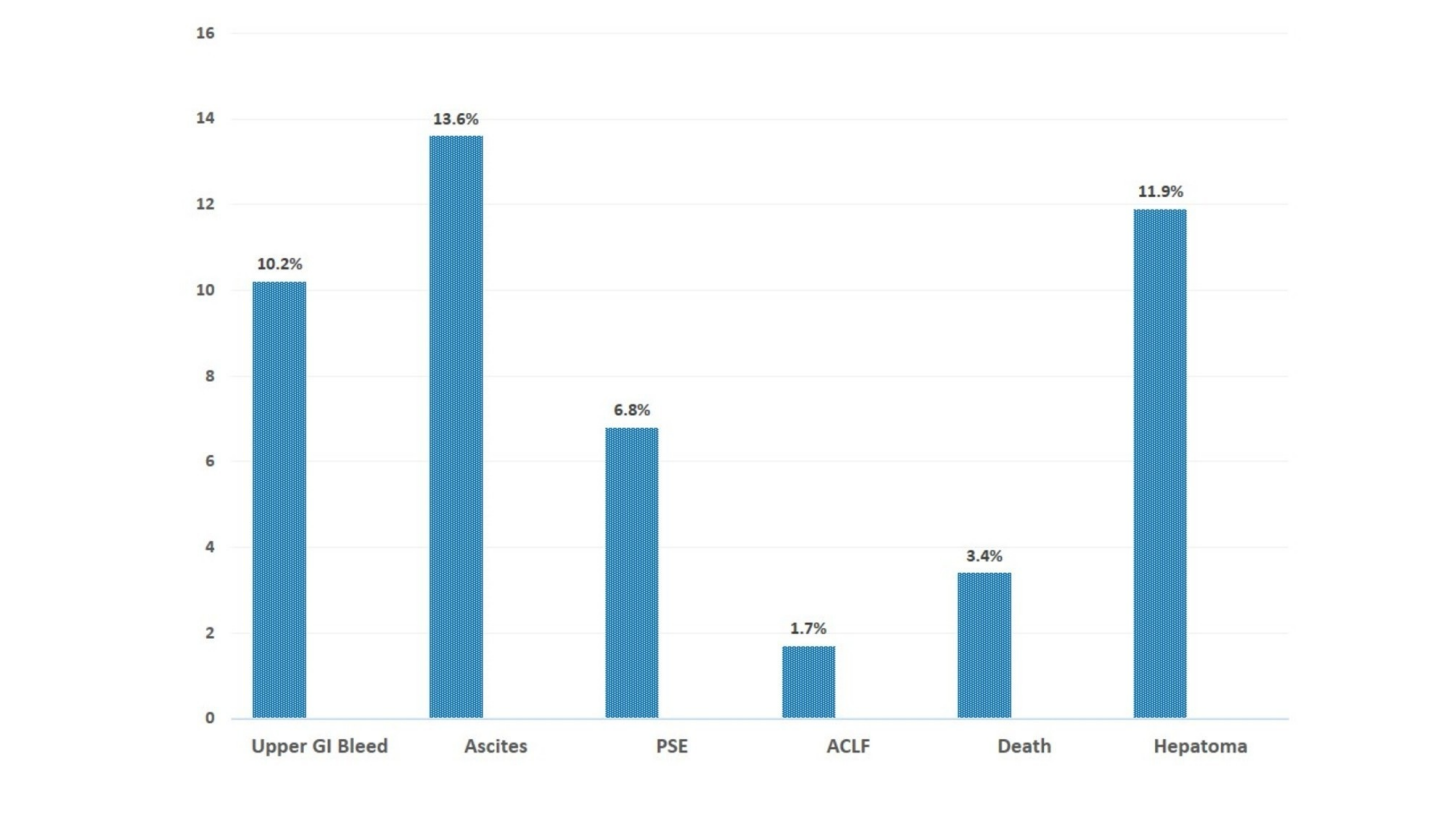
Complication due to cirrhosis PSE, portosystemic encephalopathy; ACLF, acute on chronic liver failure

The new development of complications because of cirrhosis was noted in the decompensated group, as presented in Figure 2. Ascites was found to be in 13.60%, followed by hepatoma at 11.90%, upper gastrointestinal (GI) bleed in 10.20%, portosystemic encephalopathy in 6.80%, acute on chronic liver failure in 1.70%; 3.40% deaths were also observed.

## Discussion

Cirrhotic patients should be prioritized for treatment per AASLD guidelines [11]. Our study comprised patients with compensated and decompensated cirrhosis due to HCV GT-3. In this study, SVR 24 was achieved in 32 (88.8%) of compensated treatment-naïve patients compared to treatment-naïve in decompensated in which 27 (84.4%) patients achieved SVR 24. Whereas treatment-experienced compensated and decompensated patients achieved SVR 24 in eight (90%) and 13 (81.2%), respectively. In the TARGET study on GT-3, SVR12 was achieved in 58% of treatment-naïve cirrhotic patients while in treatment-experienced patients with cirrhosis, viral clearance was 42% [12]. A study by Jacobson et al. on genotype 2 and 3 revealed a low response rate in GT-3 and an even more decreased response in case of cirrhosis [13]. Zeuzem et al. demonstrated 68% SVR12 with sofosbuvir and ribavirin in patients with cirrhosis [9], which closely resembles our study. The differences in the outcomes of clinical trials reflect the change in clinical practice and inclusion criteria in these studies.

In patients with cirrhosis, it is the decompensation of liver disease and previous treatment exposure that further decreases the chances of viral clearance in our study. In another study by Foster et al., which included 408 cirrhosis patients with decompensation due to HCV GT-3, viral clearance was 68.8% with sofosbuvir and ribavirin treatment [14].

In terms of viral clearance, the number of studies in the past showed age and gender disparity after Interferon therapy. In one study by Belci et al. [15], there was a significantly higher SVR in females aged ˂50 years. Likewise, Bhattacharya et al. found a higher SVR in premenopausal women [16]. In contrast, our study showed no statistically significant difference based on age and gender in patients with compensated and decompensated cirrhosis.

The reason behind the reduced response to treatment in cirrhosis is not completely understood but is likely multifactorial [17]. Data from cirrhotic liver patients suggests that SOF is taken up effectively and phosphorylated into its active compound by hepatocytes [17]. The shunting of blood because of raised portal pressure may affect local drug concentration and cirrhosis, on its own, effects immunity, which are important factors for viral clearance in patients with DAA treatment [17]. Younossi and colleagues found that patients with GT3 infection had lower levels of products from late in cholesterol synthesis [18]. These pieces of evidence support the hypothesis that the unique effects on lipid metabolism caused by HCV GT-3 may affect antiviral response, which is more pronounced in cirrhosis.

SOF and RIB in combination are safe in patients with cirrhosis. During the course of treatment, patients with decompensation experienced more adverse events in comparison to compensated cirrhosis. A great majority of patients came up with fatigue followed by myalgia, nausea, headache, pruritis, nausea, and nasal congestion. However, all these were managed easily in the outpatient setting except for two patients with ribavirin-related anemia who were managed by dose reduction and transfusion.

Not unexpectedly, the population of advanced liver disease patients is prone to develop serious adverse events, mainly related to end-stage liver disease. In this study, new onset of ascites, upper gastrointestinal bleeding, and portosystemic encephalopathy was found. Three patients developed hepatoma and one developed acute on chronic liver failure and died. The cause of the second death was not determined.

Due to the suboptimal response with SOF and RIB in compensated and decompensated cirrhosis in GT-3 HCV, research should be done on new DAAs, especially cirrhotic patients in this part of the world.

## Conclusions

Sofosbuvir in combination with ribavirin is safe but suboptimal in treatment outcome, particularly in treatment-experienced patients with decompensated cirrhosis than in treatment-naive patients with compensated cirrhosis due to HCV GT-3.
